# Do remote clinical trials offer carbon savings while maintaining research quality? Experiences from the Eczema Bathing Study

**DOI:** 10.1093/skinhd/vzaf122

**Published:** 2026-02-18

**Authors:** Vaibhav Chaganti, Emma L Campbell, Jessica Griffiths, Amanda Roberts, Lisa Fox, Kim S Thomas

**Affiliations:** Centre of Evidence Based Dermatology, School of Medicine, University of Nottingham, Nottingham, UK; Centre of Evidence Based Dermatology, School of Medicine, University of Nottingham, Nottingham, UK; Clinical Trials and Statistics Unit, The Institute of Cancer Research, London, UK; Centre of Evidence Based Dermatology, School of Medicine, University of Nottingham, Nottingham, UK; Clinical Trials and Statistics Unit, The Institute of Cancer Research, London, UK; Centre of Evidence Based Dermatology, School of Medicine, University of Nottingham, Nottingham, UK

## Abstract

**Background:**

Traditional clinical trials contribute significantly to the carbon footprint of healthcare. They are also expensive and take a long time to complete. Remote trials could offer part of the solution. They are a type of decentralized trial that use digital innovations to move all activities to participants’ homes or online. This gives them the potential to speed up recruitment, reduce overall trial duration, improve geographical inclusion and reduce the trial’s carbon footprint.

**Objectives:**

To assess whether a remote trial could reduce a trial’s carbon footprint, while maintaining the research quality needed to answer the scientific questions. The primary objective was to calculate the carbon footprint of a remote eczema trial and compare it with traditional and hybrid trials; the secondary objective was to assess quality indicators of remote, hybrid and traditional eczema trials in terms of speed of recruitment, retention and adherence to interventions.

**Methods:**

Carbon footprinting of the remote Eczema Bathing Study (*n* = 438, 4 weeks’ duration) was completed using the National Institute for Health and Care Research (NIHR)-funded detailed guidance and method to calculate the carbon footprint of a clinical trial. This footprint was compared with footprints of previously published publicly funded trials. Recruitment, retention and adherence metrics were compared between the Eczema Bathing Study and eight other NIHR-funded eczema treatment trials.

**Results:**

The carbon footprint of the Eczema Bathing Study was 14 tonnes CO_2_e. The most carbon-intensive activity was staff commuting. The mean speed of recruitment for remote, hybrid and traditional trials was 52, 27 and 13 participants per month, respectively. Mean retention for remote, hybrid and traditional trials was 90%, 95% and 95%, respectively. Adherence was defined individually for each study, making comparison difficult.

**Conclusions:**

The Eczema Bathing Study’s lower carbon footprint in comparison with the other clinical trials mentioned in this study was largely due to online recruitment methods, reduced need for patient and staff travel, and reduced overall trial duration. Adherence and retention were slightly lower than for traditional trials but remained within predefined limits for the study’s size calculations. In situations where remote assessment of diagnosis and outcomes is possible, and where advertising direct to participants is appropriate, running trials completely online has the potential to improve speed of recruitment, reduce overall trial duration and reduce carbon emissions.

## Teaser

Using the NIHR-funded detailed guidance and method to calculate the carbon footprint of a clinical trial, we calculated the footprint of the fully remote Eczema Bathing Study. This study had a low footprint, largely due to online recruitment methods, reduced need for patient and staff travel, and reduced overall trial duration. Our findings highlight adaptations to trial design and delivery that we urge other researchers to consider so that future trials can be run in a lower carbon manner.

What is already known about this topic?

- Clinical trials contribute significantly towards the carbon footprint of healthcare.
- Decentralized clinical trials are becoming an increasingly popular way of driving efficiencies, but little is published about their carbon footprint.
- Twelve traditional trials footprinted in 2009 showed a mean carbon footprint of 78.4 tonnes CO_2_e (range 42.1–112.7).
- Ten publicly funded clinical trials recently footprinted by the Greener Trials Group showed a range of 16–765 tonnes CO_2_e per trial.

What does this study add?

- The carbon footprint of the Eczema Bathing Study was 14 tonnes CO_2_e.
- The reduction in carbon footprint for this remote trial was largely driven by faster recruitment, reduced need for patient and staff travel, and reduced overall trial duration.
- Emissions from staff commuting contributed most to the carbon footprint of this remote trial.
- Future trialists can use these results to inform lower carbon choices when designing and conducting trials.

Clinical trials are the gold standard for establishing whether a particular intervention is suitable for patients.^1^ There are different trial designs. Decentralized clinical trials (DCTs) use digital innovations to move trial activities to participants’ homes, reducing or eliminating physical visits to trial centres. The term DCT includes studies that are fully online (remote trials) and hybrid trials. Remote trials have no in-person interaction between participants and healthcare professionals. Hybrid trials use remote methods in combination with more traditional, site-based methods.^2^ In more traditional trial designs, all study visits and assessments are conducted in person.

Remote trials are becoming increasingly popular. They can be cheaper than traditional trials, the cost of which can run into millions of pounds.^3^ Remote trials also have the potential to recruit from wider geographical areas, thereby improving accessibility.^4^ Furthermore, recruitment is one of the biggest issues facing successful trial delivery and often takes longer than expected, delaying trial completion and leading to increased costs. A paper published in 2017 looked at 151 randomized controlled trials (RCTs) funded by the National Institute for Health and Care Research (NIHR) and found that the recruitment target was achieved in only 56% of trials.^5^ Remote trials avoid the need for recruiting sites and participant travel, thereby potentially improving speed of recruitment through increased accessibility. In addition, the growth in patient-reported outcome measures and remote assessment tools makes remote trials more feasible. However, remote trials do have limitations. Firstly, it is more difficult to confirm diagnosis and provide clinical support in the absence of face-to-face assessments. Secondly, some patients may face digital exclusion. Finally, remote trials may experience lower retention and adherence as limited contact with research and clinical teams could lead to reduced engagement. Poor retention makes it difficult to interpret results if there is differential drop out between treatment arms, and poor adherence may lead to underestimation of the true efficacy of an intervention.^6,7^

Carbon footprinting of clinical trials is a relatively recent endeavour, following calls by funders and governments to reduce the environmental impact of research. In 2007, the Sustainable Clinical Trial Group conducted a carbon audit of the Corticosteroid Randomisation After Significant Head injury (CRASH) trial and found that it contributed significantly to greenhouse gas emissions.^8,9^ Another paper published in 2009 calculated the carbon footprint from a sample of pragmatic RCTs and found the mean emissions per trial were 78.4 tonnes CO_2_e (range 42.1–112.7).^10^ In response to these findings, the NIHR produced guidelines outlining where research design can help to reduce research waste and carbon emissions.^11^ Subsequently, members of the Medical Research Council–NIHR Trials Methodology Research Partnership Greener Trials Group developed the NIHR-funded detailed guidance and methodology to calculate the carbon footprint of clinical trials.^12^ This is continuously updated as the Greener Trials Group get more data from trials that involve different interventions, activities and therapeutic areas. To the best of our knowledge, no studies have yet published the impact on the carbon footprint of delivering trials remotely, or of any eczema or dermatology RCTs.

This project aimed to calculate the carbon footprint of a fully remote trial while considering quality metrics in terms of speed of recruitment, retention and adherence to ­interventions.

The primary objective was to carbon footprint a remote eczema trial and compare this with previously footprinted traditional and hybrid trials, identify carbon hotspots and assess how future trials could reduce carbon emissions.

The secondary objective was to compare metrics of remote, hybrid and traditional eczema trials in terms of (i) speed of recruitment, (ii) retention and (iii) adherence to interventions.

## Materials and methods

### Data sources

The Eczema Bathing Study (*n* = 438, 4 weeks’ duration) was the remote trial to be carbon footprinted.^13^ This was conducted at the Centre of Evidence Based Dermatology (CEBD) with support from the Nottingham Clinical Trials Unit, and sponsored by Nottingham University Hospitals National Health Service Trust.^14^ The Eczema Bathing Study belongs to a series of remote trials as part of the Rapid Eczema Trials project.^15^ Ten trials that were previously footprinted using the same methodology (the NIHR-funded detailed guidance and methods) were used for context.^16^

Recruitment, retention and adherence were compared between the Eczema Bathing Study and eight NIHR-funded, UK eczema treatment trials (two remote, one hybrid, five traditional) as reported in the recent NIHR evidence summary.^17^ Data for the comparison were sourced from published trial reports, internal documents and interviews with trial staff.

The eight comparator trials were:

- Best Emollient for Eczema (BEE)^18^
- Bath Additives for the Treatment of Eczema in Children (BATHE)^19^
- CLOthing for the Relief of Eczema Symptoms (CLOTHES)^20^
- Softened Water Eczema Trial (SWET)^21^
- TREatment of severe Atopic eczema in children Taskforce (TREAT)^22^
- Children with Eczema, Antibiotic Management (CREAM)^23^
- Eczema Care Online 1 (ECO 1)^24^
- Eczema Care Online 2 (ECO 2)^24^

The Barrier Enhancement for Eczema Prevention (BEEP) trial was excluded as it was an eczema prevention study that recruited pregnant mothers at high risk of having a child with atopy rather than people with eczema.^25^

### Study design

#### Carbon footprinting the Eczema Bathing Study

A carbon footprint is a measure of greenhouse gases produced by an activity, quoted in kg or t CO_2_e (1 tonne = 1000 kg). It is calculated by multiplying activity data by emission factors. For this study, an ‘activity’ is anything that occurs in the lifetime of a clinical trial. An emission factor is a coefficient that allows conversion of activity data into greenhouse gas emissions.

The Eczema Bathing Study was conducted between 1 January 2023 and 7 November 2024. This marked the time between the start of trial design and the sharing of trial results. Carbon footprints of activities were calculated using the NIHR-funded detailed guidance and method to calculate the carbon footprint of a clinical trial.^12^ This methodology defines the scope of what are considered to be clinical trial-specific activities contributing to the total carbon footprint. V.C. and E.L.C. attended the Greener Trials Clinical Trial Carbon Footprinting Drop in Clinics run by J.G. and L.F. for training in carbon footprinting and application of the guidance.^26^ Where activities included the Eczema Bathing Study were not covered by existing guidance, or more up-to-date emission factors had been released, J.G. and L.F. advised on the emission factors and calculations to use, resulting in updates to the guidance.

The modules and activities footprinted in the Eczema Bathing Study were documented using the ‘Enabling Lower Carbon Clinical Trials Quick Guide and Data Collection Tool’.^26^ This resource displays the 10 modules described in the detailed guidance and all their associated clinical trial processes, within which different activities occur. Certain modules were not applicable to the Eczema Bathing Study (Table 1).

**Table 1:** Carbon emissions of each activity footprinted in the Eczema Bathing Study

Modules	Activity being footprinted	Carbon footprint (kg CO_2_e)
**1. Trial set up, promotion and recruitment (see module 1 and Tables S1–S5 in Supporting Information)**		
1.1 Production of trial documentation to be sent to sites/participants	Posters and leaflets producedLetter invites produced	13.75 (0.10%) LOW
1.2 Provision/postage of trial documentation	Postage of posters and leaflets from NCTU to GPs	17.87 (0.13%) LOW
	Postage of invitation letters from GPs to participants	
1.3 Online recruitment	Text messages sent from GPs to participants	50.45 (0.36%) MEDIUM
	Paid Facebook adverts	
	Newsletters	
1.4 Generation and storage of online intervention materials	Survey to inform intervention development	0.35 LOW
1.5 Webpages	Webpages containing videos, images, PDFs and links to other pages	12.24 LOW
**2. CTU emissions (see module 2 and Tables S6, S7 in Supporting Information)**		
2.1 Energy consumption at CTU (electricity)	Energy consumption per square metre of air-conditioned office space attributed to electricity	585.10 (4.18%) MEDIUM
2.2 Energy consumption at CTU (heating)	Energy consumption per square metre of air-conditioned office space attributed to heating (natural gas).	1205.18 (8.6%) HIGH
2.3 Homeworking energy consumption (heating and electricity)	Energy consumption attributed to staff working from home	1676.24 (11.97%) HIGH
2.4 Trial team commuting	Travel of staff	10 340.23 (73.8%) HIGH
**3. Trial-specific meetings and travel (see module 3 and Table S8 in Supporting Information)**		
3.1 Travel to meetings	Online meetings on Microsoft Teams	187.13 (1.33%) MEDIUM
	One, in-person meeting in London	
3.2 Sustenance	Lunch provided at London meeting	18.20 (0.13%) LOW
**4. Intervention (not applicable)**		
**5. Data collection (see module 5 in Supporting Information)**		
5.1 Data collection and exchange	Emails in the Rapid Eczema Trial mailbox	185.01 (1.32%) MEDIUM
	Data collection via REDCap trial database	
	Electronic questionnaires completed by participants	
**6. Trial supplies and equipment (not applicable)**		
**7. Trial specific assessments (not applicable)**		
**8. Samples (not applicable)**		
**9. Laboratory (not applicable)**		
**10. Trial close out (see module 10 in Supporting Information)**		
10.1 Storage and archiving of essential trial documentation and data	Storage of electronic documents in Microsoft Teams channels	2.39 LOW

To aid visualization, carbon emissions are labelled as HIGH (more than 1000 kg CO_2_e), MEDIUM (50–1000 kg CO_2_e), and LOW (less than 50 kg CO_2_e). CTU, Clinical Trial Unit; GP, general practitioner; NCTU, Nottingham Clinical Trials Unit.

Primary activity data were collected where possible. Secondary data were used when the exact quantity of the activity taking place could not be determined or measured. This either took the form of a mean amount or adopted the value of previously published and recommended activity data. See the Supporting Information, including Tables S1–S9, for a full breakdown of the footprinting calculations, clarification of whether primary or secondary activity was used for each activity, and acknowledgement of any assumptions that were made. Quality assurance of the carbon footprint calculations was completed by the Greener Trials Group (J.G.) and CEBD staff (E.L.C.).

#### Comparing metrics between the Eczema Bathing Study and other eczema trials

##### Speed of recruitment.

Speed of recruitment (participants per month) was defined as:


$$\frac{\mathrm{the number of participants enroled in the study}}{\mathrm{number of months in the period of recruitment}}$$

##### Retention.

Retention was defined as:


$$\frac{\begin{array}{l} \text{the number of participants who were included in}\\ \text{primary outcome analysis prior to any imputation} \end{array}}{\text{total number of patients recruited}}$$

##### Adherence.

Adherence was defined individually for each study, focusing on adherence across the entire trial period rather than adherence at specific timepoints.

The number and percentage of participants who were adherent with allocated interventions was presented with the following subset of participants:

- Participants who provided adherence data:
  $$\frac{\begin{array}{l} \text{the number of participants who were adherent from}\\ \text{participants who provided enough data} \end{array}}{\text{total number of participants who provided enough data}}$$
- All participants enrolled onto the study. Assumptions were made for participants with missing adherence data:
  $$\frac{\begin{array}{l} \mathrm{number of participants who were adherent with}\\ \mathrm{imputation using assumptions made for missing data} \end{array}}{\mathrm{total number of patients recruited}}$$

### Statistical analysis

This was an exploratory secondary analysis focused on descriptive statistics and quantitative findings. The calculations were done using Microsoft^®^ Excel^®^ (Version 16.100.3).

### Patient and public involvement

The Eczema Bathing Study was co-designed with people with eczema.^27^ The current project was discussed with members of the CEBD’s patient panel, involving nine people with eczema. Panel members helped to identify key areas in trials where researchers’ decisions could impact on carbon footprint:

- Mode of transport taken to work
- Homeworking vs. office working
- Online meetings vs. face-to-face meetings
- Cameras on vs. cameras off during online meetings
- Letters vs. texts for recruitment
- Website with videos vs. without videos

Carbon footprinting data were used to see which of these choices were carbon intensive and to inform our recommendations to help trial teams choose how to conduct future trials in a lower-carbon manner.

## Results

### Carbon footprint of the remote Eczema Bathing Study

The total carbon footprint of the Eczema Bathing Study was 14 t CO_2_e (carbon intensity = 31.96 kgCO_2_e per participant recruited). The largest hotspot was trial team commuting (10 t CO_2_e), which was responsible for the majority of the study’s carbon footprint. The second main contributor was energy consumption (heating and electricity), both for office working (2 t CO_2_e) and home working (2 t CO_2_e). All of these activities fall under ‘Clinical Trial Unit (CTU) emissions’ (Table 1).

In the Eczema Bathing Study, some activities were carried out in more than one way. For example, the study included both face-to-face and online meetings, and in the online meetings, some people had cameras on and others had cameras off. Recruitment ‘mailshots’ included some text messages and some printed letters. Furthermore, some of the public-­facing webpages contained videos, whereas others did not. To help researchers assess how activities could be modified to reduce the environmental impact of future clinical trials, we compared the carbon footprints of these variations (Table 2).

**Table 2:** Choices for reducing the carbon footprint of clinical trials: implications of decisions around trial design and delivery

Activity choice	Most carbon intensive	Least carbon intensive
Type of meeting	Face to face (in London):	Online:
	kg CO_2_e for one meeting = 129.9 (or 52 per hour of meeting)	Mean kg CO_2_e per meeting = 3.58 (or 2.63 per hour of meeting)
Cameras on or off during online meeting	Cameras on:	Cameras off:
	0.1573 kg CO_2_e per person per hour	0.0063 kg CO_2_e per person per hour
Type of mailshot	Letters:	Text messages:
	21.44 kg CO_2_e for 1411 letters (or 15.19 g per letter)	0.47 kg CO_2_e for 33 411 text messages (or 0.01 g per text)
Website with videos or without videos	With videos:	Without videos:
	Mean CO_2_e for pages: 1.52 kg CO_2_e, plus 0.18 kg CO_2_e for watching the videos	Mean CO_2_e for pages: 0.011 kg CO_2_e, plus 0.05 kg CO_2_e for reading the text on the page
	1.72 kg total mean	0.061 kg total mean

Details of the activities, calculations and source materials are provided in the Supporting Information (Section 3.1, Table S8 and the Supporting Information reference list).

To help put the carbon footprint of the Eczema Bathing Study into context, we considered data from 10 traditional or hybrid trials that have previously been footprinted using the same methodology (Table 3).^16^ The remote Eczema Bathing Study had the lowest total carbon footprint (14 t CO_2_e, compared with a range of 16–765 t CO_2_e). CTU emissions were the top carbon hotspot in the Eczema Bathing Study and also in the top 3 hotspots for 9 of the 10 trials. Four of the 10 trials had CTU emissions as their main hotspot.

**Table 3:** The details, carbon footprint and carbon hotspots of 10 randomized controlled trials previously footprinted using the National Institute for Health and Care Research-funded detailed guidance and method to calculate the carbon footprint of a clinical trial

Trial name	Countries (*n*)	Sites (*n*)	Participants (*n*)	Trial duration (years)	Total carbon footprint (t CO_2_e)	Top three hotspot modules and their percentage contribution to the total carbon footprint
Emerge	1	1	535	6	74	2 (27%), 7 (25%), 6 (17%)
Heal Covid	4 (UK)	109	1245	4	91	5 (79%), 2 (17%), 10 (3%)
Interact-3	10	122	7064	6	765	2 (71%), 7 (8%), 3 (7%)
Interval	4 (UK)	51	2372	5.5	61	3 (46%), 7 (22%), 2 (18%)
Mavmet	1 (UK)	6	90	5	18	7 (39%), 10 (20%), 9 (15%)
On-Pace	1 (UK)	1	102	2.5	16	9 (36%), 2 (31%), 7 (16%)
Premise	3 (UK)	10	536	5	25	2 (54%), 7 (27%), 3 (11%)
ReStart	4 (UK)	122	537	8	109	2 (72%), 7 (11%), 6 (6%)
Shamrock	1	5	80	7	58	7 (41%), 9 (31%), 2 (12%)
UK Stand Together	2 (UK)	116	12 580	2.75	107	6 (49%), 2 (35%), 3 (14%)

Reproduced from Griffiths *et al*.^16^ under Creative Commons CC BY 4.0 licence. Module 2, Clinical Trial Unit emissions; module 3, trial-specific meetings and travel; module 5, data collection; module 6, trial supplies and equipment; module 7, trial-specific assessments; module 9, laboratory; module 10, trial close out.

### Speed of recruitment, retention and adherence

Speed of recruitment, retention and adherence were compared between the Eczema Bathing Study and NIHR-funded eczema treatment trials as reported in the recent NIHR eczema evidence summary (Table 4).^17^ Speed of recruitment for remote, hybrid and traditional trials was 52, 27 and 13 participants per month, respectively. Retention for remote, hybrid and traditional trials was 90%, 95% and 95%, respectively. Adherence was defined individually for each study, making direct comparison difficult (Table 4).

**Table 4:** Comparison of speed of recruitment, retention and adherence between the Eczema Bathing Study and other National Institute for Health and Care Research-funded eczema treatment trials, including remote, hybrid and traditional trials

Trial	Trial type	Speed of recruitment (participants per month)	Retention	Adherence: complete data only	Adherence: adjusting for missing data
Eczema Bathing Study	Remote	88	388/438 (89%)	202/278 (73%)	269/438 (61%)
Eczema Care Online (parents and carers) Trial	Remote	34	311/340 (91%)	NA	299/340 (88%)
Eczema Care Online (young people) Trial	Remote	34	304/337 (90%)	NA	310/337 (92%)
Bath Emollients for Eczema Trial	Hybrid	27	461/483 (95%)	392/424 (92%)	NA
Best Emollients for Eczema Trial	Traditional	26	524/550 (95%)	NA	152/550 (28%)
CLOthing for the Relief of Eczema Symptoms	Traditional	18	282/300 (94%)	102/124 (83%)	117/149 (79%)
Softened Water Eczema Trial	Traditional	13	323/336 (96%)	NA^a^	NA^a^
Treatment of Severe Atopic Eczema Trial	Traditional	3	103/103 (100%)	NA	83/103 (81%)
Children with Eczema Antibiotic Management study	Traditional	7	101/113 (89%)	NA	60/97 (62%)

Calculations for the speed of recruitment are shown in Table S9 (see Supporting Information). NA, Not available. ^a^Household intervention, so individual adherence not applicable.

## Discussion

The carbon footprint of the Eczema Bathing Study was 14 t CO_2_e. This low footprint was largely due to online recruitment, the absence of patient travel, low staff travel and shorter overall trial duration. This total carbon footprint was much smaller than the trials footprinted in 2009 (range 42.1–112.7 t CO_2_e; mean 80.65 t CO_2_e).^10^ It was also smaller than the footprints of other trials calculated using the same methodology (range 16–765 t CO_2_e; mean 68 t CO_2_e).^16^ It is important to note that equitable comparisons of the carbon footprints of these clinical trials was not possible due to the diversity of the trial interventions. Data from the other trials are included here to provide context when considering the carbon footprinting data from the Eczema Bathing Study. For all of these trials, CTU emissions were consistently a major hotspot, including energy consumption for electricity and heating, and staff commuting. Carbon emissions of homeworking vs. office-working were similar, although it should be noted that for many organizations, offices will still be lit and heated or cooled daily, irrespective of whether or not staff are working from home.

In this project, we found that the carbon footprint of some activities could be changed by making small adaptations. For example, including videos on webpages increases the carbon footprint. However, it is possible that these may increase engagement and improve recruitment. Similarly, running online meetings with cameras off generated a lower carbon footprint than meetings with cameras on. However, having cameras off may affect interaction and engagement. It was not possible in this study to attribute the carbon footprint of recruitment to the specific method of general practitioner (GP) communication with patients (text or letter) as recruitment by GP practice was not recorded. However, producing and sending study invites by letter is more carbon intensive than sending them by text message (15.19 g CO_2_e vs. 0.01 g CO_2_e).

Recruitment was fastest in the remote eczema treatment trials compared with traditional and hybrid eczema trials, with the Eczema Bathing Study recruiting the fastest. This was probably due to the wider geographical accessibility enabled by online recruitment methods. The Eczema Bathing Study was also part of a wider citizen science project, which may have helped to further boost study recruitment. Recruiting more quickly than usual had a significant impact on carbon footprint by reducing the trial’s overall duration and, therefore, CTU emissions.

Retention in the three remote eczema trials was slightly lower than observed in traditional and hybrid eczema trials but was within predefined limits for the size calculations for these studies. It is possible that the limited contact participants have with research and clinical teams in remote trials reduces engagement. This is a risk as poor retention makes it difficult to interpret results if there is a differential drop out between treatment arms. However, it is of note that in a recent smoking cessation trial, retention was higher among participants recruited online compared with those recruited in person.^28^ Interestingly, in a recent review exploring the impact of digital tools on the delivery of central nervous system trials, the authors discuss how use of these tools can potentially improve study retention and adherence.^29^

In 2019, the NIHR published carbon reduction guidelines, encouraging all research teams to consider carbon implications when designing clinical studies. Furthermore, in 2024, the UK research and innovation sector co-developed an environmental sustainability concordat, representing a shared ambition for the UK to deliver cutting-edge research in a more environmentally responsible and sustainable way.^30^ Signatories of this concordat include many universities, research institutes and funding bodies, including the NIHR. By reporting their efforts to reduce the carbon footprint of clinical trials, researchers will be aligning with the commitments of concordat signatories.

The NIHR-funded detailed method and guidance to carbon footprint clinical trials includes an inventory of clinical trial activity data and emission factors, which are constantly updated. The more trials footprinted using this method, the more activities, interventions and emission factors are characterized, and the more robust and widely applicable the guidance becomes. Carbon footprinting of the Eczema Bathing Study was the first time a fully remote trial and an eczema trial have been footprinted using this method, and so this work has provided new activities for inclusion in the NIHR-funded guidance. The Greener Trials team has recently been awarded funding from Wellcome to develop a Greener Trials Toolkit. This will be an online, free-to-use, open science clinical trial carbon footprinting calculator, with inbuilt links to hotspot mitigation guidance plus an accumulating database of clinical trial carbon footprinting data.

This remote trial was footprinted using best practice guidance and with ongoing support and quality checks from the Greener Trials Group. Primary activity data were used for the calculations wherever possible, and where assumptions were made or secondary data used, they have been transparently reported in the Supporting Information.

Remote trials offer a potential approach to reducing the carbon footprint of clinical research. However, it is important to note that direct comparison of carbon footprint between trials is difficult, as they are of different designs and sizes, and include different patient populations and treatment approaches. The Eczema Bathing Study is unique in the fact that, to the best of our knowledge, it is the first fully remote trial or dermatology trial that has been carbon footprinted.

Finally, carbon footprinting the trial only tells us about greenhouse gas emissions. There are other factors associated with running a clinical trial such as water use, land use and waste, which also affect the environment.

Remote trials can reduce carbon footprint by increasing speed of recruitment, which in turn reduces overall trial duration, and by removing emissions generated by recruiting sites and participant travel. CTU emissions, incorporating heating, electricity and commuting, are likely to be a major contributing factor to the carbon emissions of remote clinical trials. Although reducing energy consumption can be difficult, host institutions should implement policies and practices to reduce building emissions. They should also take the lead in incentivizing and enabling staff to use more carbon-friendly commuting methods wherever possible.

Removing or reducing the need for patients and staff travel for consent, randomization, follow-up and assessments could significantly reduce the carbon footprint of clinical trials. Smaller factors also come in to play during remote trial delivery, such as how online meetings are run (including whether they are recorded and whether artificial intelligence note-takers are used), what type of content is put on the study website and the methods used to contact patients.

It is important to recognize that not all trials can be conducted entirely online. Remote trials may not be appropriate if the diagnosis needs to be confirmed by a clinician prior to joining the study, if there are safety concerns requiring in-person surveillance, tests and clinical assessments, or if the trial intervention must be administered in person. If it is challenging to blind intervention allocations, patient-­reported outcomes may be less appropriate, although opportunities to monitor health data and activities remotely may help in this regard. In addition, it is important to bear in mind that potential benefits for patients may be removed by moving trial activities online, for example access to healthcare professionals.

Furthermore, it is important to exercise caution when ‘comparing’ the carbon footprints of clinical trials. There is currently no international consensus on the best approach for comparing carbon footprints of clinical trials. Reaching such a consensus is complicated as many trial-specific factors contribute to carbon footprinting data. Measuring carbon intensity could provide a way forward in some instances, although caution is still needed as it has been shown that this figure can discriminate against rare disease trials.^12^ As this field of research moves forward, it will become increasingly important to find agreed methodology that enables equitable comparison between diverse trials.

In this study, we have shown that running a trial remotely can significantly shorten the overall duration of a trial and reduce carbon footprint. Other researchers considering this approach should be urged to evaluate all aspects of trial design and delivery, weighing up which activities are necessary to maintain study quality and participant engagement, and which can be removed or adapted to be more environmentally friendly.

## Supplementary Material

vzaf122_Supplementary_Data

## Data Availability

All data for this project were provided at the time of submission as Supplementary Information. All materials relating to the design and conduct of the Eczema Bathing Study are available at https://figshare.com/authors/Rapid_Eczema_Trials/20938393.
