# Supplementary material for: Do remote clinical trials offer carbon savings while maintaining research quality? Experiences from the Eczema Bathing Study
Source: Skin Health Dis. 2026 Feb 18;6(3):283–90. doi: 10.1093/skinhd/vzaf122 (PMC13220035; doi:10.1093/skinhd/vzaf122)
Supplement: vzaf122_Supplementary_Data [file vzaf122_supplementary_data.docx]

# **Supplementary Information**

This Supplementary Information document, including tables S1-S9, provides a detailed description of how the carbon footprint of each activity was calculated. Primary data was used wherever possible. When secondary data has been used, this is made clear, along with detail of any assumptions that were made. Information sources relating to the emission factors and secondary data used are referenced after each individual emission factor. The MRC-NIHR Trials Methodology Research Partnership (TMRP) Greener Trials Group update the guidance annually to include new or better emission factors if these have become available since the last update. To access the most up-to-date emission factors, it is therefore recommended to visit the Greener Trials website. (1)

Abbreviation: EF - emission factor; NCTU – Nottingham Clinical Trials Unit

Module 1

1.1 Production of trial documentation to be sent to sites/participants

The trial documents produced and printed were:

- Coloured 1-page posters (A4) and leaflets (A5) which would be posted to GP surgeries (participant identification centres).
- Coloured 1-page A4 letter invites which would be sent from GPs to participants.

Carbon footprint of producing and printing the paper:

Number of pages x weight of paper (kg) x (paper manufacture EF [0.91048 kg CO_2_e per kg of paper] (2) + colour printing EF [0.31786 kg CO_2_e per kg of paper]) (3).

Secondary data:

- A4 paper weighs 0.005 kg
- A5 paper weighs 0.0025 kg

Assumptions:

- The number of posters and leaflets printed was the same as the number posted.

Table S1 shows the number of A4 posters and A5 leaflets sent to each GP surgery, and the distances between the NCTU and each GP surgery. Table S2 shows the number of A4 letter invites printed and sent from GPs to participants.

A4 posters = 219

A5 leaflets = 1217

A4 letter invites = 1411

Total number of A4 papers = 219 + 1411 = 1630

Total number of A5 papers = 1217

Carbon footprint of producing and printing paper:

1630 x 0.005 kg = 8.15 kg

1217 x 0.0025 kg = 3.0425 kg

8.15 + 3.0425 = 11.1925 kg (total weight of paper)

11.1925 x (0.91048 + 0.31786) = **13.75 kg CO_2_e**

1.2 Provision/postage of trial documentation

The trial documents posted were:

- Posters and leaflets sent from NCTU to GP surgeries.
- Letter invites sent from GPs to participants.

Carbon footprint for postage of trial materials by post:

Total weight of the materials (in tonnes) x mean distance travelled (kilometres) = tonne.km (t.km)

t.km x road freight EF (0.19443 per t.km). (3)

Secondary data:

- A4 paper, including an A4 envelope, weighs 0.005 kg
- A5 paper weighs 0.0025 kg
- The distances between GP surgeries and participants are unknown. Our calculations instead used the mean distance between GP and patient in the UK, which is 3.3 km (4).

Assumptions:

- When posters/leaflets were sent to GP surgeries, there was also an A4 paper envelope per surgery.
- When letter invites were sent to participants, there was an A4 paper envelope per participant.

Table S1 shows the number of A4 posters and A5 leaflets sent to each GP surgery, and the distances between the NCTU and each GP surgery. Table S2 shows the number of A4 letter invites printed and sent from GPs to participants.

Number of A4 posters = 219

Number of A5 leaflets = 1217

Number of A4 envelopes (given by number of GP surgeries which received posters; some of these also received leaflets in the same envelope) = 76

Weight:

A4 posters: 219 x 0.005 = 1.095 kg

A5 posters: 1217 x 0.0025 = 3.0425 kg

A4 envelopes = 76 x 0.005 = 0.38 kg

Total delivery weight = 1.095 + 3.0425 + 0.38 = 4.5175 kg = 0.0045175 tonnes

Distance between printing shop (LS11 5AS) and NCTU (NG7 2RD) = 115.873 km

Total distance between NCTU and the GP surgeries = 5690.59933km

Total distance travelled (printers to NCTU to GP) = 115.873 + 5690.59933 = 5806.47233 km

0.0045175t (weight) x 5806.47233 (distance) = 26.2307388 t.km

26.2307388 x 0.19443 EF = 5.10 kg CO_2_e (carbon footprint of postage of posters)

Number of GP invitation letters sent (A4) = 1411

Number of A4 envelopes = 1411

Total number of pages of A4 paper = 2822

Weight of materials = 2822 x 0.005 kg = 14.11 kg = 0.0141 tonnes.

Weight x mean distance from GP to patient x number of patients an invite is sent to:
0.0141 tonnes x 3.3 km x 1411 = 65.65383 t.km

65.65383 x 0.19443 EF = 12.77 kg CO2e (carbon footprint of the letter invites)

**Total carbon footprint of posting posters and invites = 17.87 kg CO_2_**

1.3 Online recruitment

Online recruitment was achieved via text messages sent from GPs to patients, paid Facebook ads, and online newsletters.

Carbon footprint of text messages:

Number of text messages sent x text message EF (0.014 g CO_2_e per SMS text message) (5).

Carbon footprint of text messages sent to patients as a recruitment tool:

See Table S3 for information on number of text messages sent.

Number of text messages sent = 33411

33411 x 0.014 = 467.754 g CO2e = **0.47 kg CO_2_e**

Carbon footprint of paid Facebook ads:

1. Carbon footprint of advertisement storage on Facebook servers/data centres
2. Carbon footprint of data transmission when people view the ad.

Assumptions:

- All Facebook ads were viewed using smartphones.
- Carbon footprint of browsing the ads on user devices is not accounted for; we estimate that people would only have spent a few seconds viewing the ad.
- Carbon footprint of Facebook servers/data centres is assumed to be 0 CO_2_e as Meta’s data centres are powered by 100% renewable energy. Note, although this figure is acceptable under the GHG Protocol, we caution that there might be carbon cost related to infrastructure, transmission and maintenance.
- The Facebook Ad Centre does not provide details on the GB size of the full ad, so we calculated the size of the ads based on the file size of any images or videos used in the ads.

Table S4 details the Facebook ads.

Carbon footprint of data transmission: For each ad, number of views x file size (GB) was calculated and the individual figures then summed (2615.24252 GB). The total is then multiplied by the data transmission EF (0.01478 kg CO_2_e per GB) (6).

2615.24252 x 0.01478 = **38.65 kg CO_2_e**

Carbon footprint of newsletters:

Two newsletters promoted study recruitment.

1. Sent on Monday 29^th^ January, to 385 recipients (69.6% of recipients opened the email) 
   <https://mailchi.mp/5d1dfcb66b3c/rapid-eczema-trials-community-newsletter-may-6164627?e=449bb22e79>
2. Sent on Monday 29^th^ April, to 643 recipients (59.5% of recipients opened the email) 
   <https://mailchi.mp/nottingham/rapid-eczema-trials-community-newsletter-february2024-8262851?e=449bb22e79>

Two components contributed to the carbon footprint of each newsletter:

1. Number of participants emailed with each newsletter x EF for emails without attachments (0.01 kg CO_2_e per email) (7).
2. The g CO_2_e per pageview for each page (produced via the website carbon calculator [**https://www.websitecarbon.com/)**](https://www.websitecarbon.com/)) multiplied by the number of views.

Newsletter A:

1. 385 x 0.01 = 3.85 kg CO_2_e
2. 2.03 g CO_2_e per view x 385 x 0.696 = 543.9588 g CO_2_e = 0.5439588 kg CO_2_e

Newsletter B:

1. 643 x 0.01 = 6.43 kg CO_2_e
2. 1.32 g CO_2_e per view x 643 x 0.595 = 505.0122 g CO_2_e = 0.5050122 kg CO_2_e

Total carbon footprint of newsletters = **11.33 kg CO_2_e**

Total carbon footprint of online recruitment = 0.467754 + 38.6532845 + 11.328971 = **50.45 kg CO_2_e**

1.4 Generation and storage of online intervention materials

During trial design, people with eczema were invited to answer some questions in an online survey that would inform intervention development.

Carbon footprint of completing the survey:

Time taken to complete all respondents’ surveys (hours) x (smartphone EF [0.015068 kg CO_2_e per hour] (8) + web surfing EF [0.009441 kg CO_2_e per hour]) (6).

Assumptions:

- Participants completed the survey using smartphones.

Number of responses = 169
Time taken to complete one survey = 5 minutes

Total time taken to complete all surveys = 845 minutes = 14.083 hours

0.009441 + 0.015068 = 0.024509 kg CO_2_e per hour (combined emission factors)

0.024509 x 14.083 hours = **0.35 kg CO_2_e (generation and storage of online intervention materials).**

1.5 Website pages

The trial website had various pages, containing a mix of videos, images, PDFs, and links to other pages.

Carbon footprint of webpages:

1. Each website’s URL was pasted into the following online carbon calculator [**https://www.websitecarbon.com/**](https://www.websitecarbon.com/). This gives the g CO_2_e per pageview for each page. This CO_2_e figure includes the presence of embedded videos, images, pdf documents, and links to other pages. This was multiplied by the number of website clicks/views.
2. Web surfing footprint for webpages with a video: number of people who viewed the video x length of video = total watch time (s). Divide by 3600 to get a watch time in hours. This was calculated individually for each webpage, then totalled. Total x (web surfing EF [0.009441 kg CO_2_e per hour] (6) + smartphone EF [0.015068 kg CO_2_e per hour kg CO_2_e]) (8).
3. Web surfing footprint for webpages without a video: the total number of clicks was multiplied by 2 minutes = watch time in minutes. This is then divided by 60 to get a watch time in hours. Multiply this by (web surfing EF [0.009441 kg CO_2_e per hour] + smartphone EF [0.015068 kg CO_2_e per hour kg CO_2_e]).

Assumptions:

- All people who viewed a video, watched the whole video.
- Each person who viewed a webpage without a video spent 2 minutes browsing the content on the page.

See Table S5 for information on webpages and calculations.

Carbon footprint of webpages:

Total g CO_2_e for all webpages combined = 10719.37 g CO_2_e = **10.72 kg CO_2_e**

Carbon footprint of web surfing (for webpages that contain videos):

52.6513889 (total watch time in hours) x (0.015068 + 0.009441) kg CO_2_e = **1.29 kg CO_2_e**

Carbon footprint of web surfing (for webpages without videos):

Total number of clicks: 283

283 clicks x 2 minutes = 566 minutes watch time

566 / 60 = 9.43 hours watch time

9.43 x (0.015068 + 0.009441) = **0.23 kg CO_2_e**

Total carbon footprint: **12.24 kg CO_2_e**

Module 2

Assumptions:

- 50% of staff time is spent working in the office and 50% is spent working at home.
- The initial Senior Trial Manager left and was replaced on 28/9/23 so the Full Time Equivalents (FTEs) of these two individuals were considered together as one. The %FTE on the bathing trial, FTE, commuting method, and distance from home to office information reflected the working pattern of the Senior Trial Manager who worked on the trial for the longest.
- Staff were footprinted for different periods depending on their role. If they were employed to design the study, they were footprinted from 01/01/2023 (or date of appointment if later) to 29/01/2024 reflecting the date of first recruitment to the study. If they were employed to deliver the trial, they were footprinted from date of appointment (01/05/23 or later) to the date when the study results were released (07/11/2024). See Table S6 for FTE information and calculations.

2.1 Energy consumption at CTU (electricity)

Carbon footprint of energy consumption (electricity) per square metre of air-conditioned office space attributed to electricity:

Office space per FTE x electricity intensity for offices per m^2^ x electricity EF (0.257 kg CO_2_e per kilowatt-hour) (2) x total FTE working on the online trial at the office.

Secondary data:

- Public sector office space is 12 m^2^ per FTE, according to the UK Employment Density Guide (9)
- The median electricity intensity for offices is 68 kWh per m^2^ for a year

12m^2^ x 68 kWh x 0.257 = 209.712 kg CO_2_e per FTE per year

Total FTE on Eczema Bathing Study = 5.58

Total FTE on Eczema Bathing Study at office = 5.58 x 0.5 = 2.79

209.712 x 2.79 = **585.10 kg CO_2_e** (in office)

2.2 Energy consumption at CTU (heating)

Carbon footprint of energy consumption per square metre of air-conditioned office space attributed to heating.

For heating, the calculation is the same as the one for electricity above. The 12m^2^ office space per person x office heating benchmark x natural gas conversion factor (0.213 kg CO_2_e per kWh) (2) x total FTE working on the online trial at the office.

Secondary data:

- Office building heating benchmark: 169 kWh/m^2^

Assumptions:

- Natural gas is the heating source.

12 m^2^ x 169 kWh/m^2^ x 0.213 = 431.964 kg CO_2_e per FTE per year

Total FTE on Eczema Bathing Study = 5.58

Total FTE on Eczema Bathing Study at office = 5.58 x 0.5 = 2.79

431.964 x 2.79 = **1205.18 kg CO_2_e**

2.3 Homeworking energy consumption

Carbon footprint associated with homeworking (includes electricity and heating):

Total number of working hours x conversion factor for homeworking (0.33378 kg CO_2_e per hour worked) (2).

Secondary data:

- 1 FTE is equal to 1800 hours (calculated based on an 8-hour working day and 225 working days per year [there are 260 working days in a year minus 35 days paid leave and sickness]).

Total FTE on Eczema Bathing Study = 5.58

Total FTE on Eczema Bathing Study working from home = 5.58 x 0.5 = 2.79

2.795 x 1800 = 5022 hours

5022 x 0.33378 = **1676.24 kg CO_2_e**

2.4 Trial team commuting

Commuting details are shown in Table S7. For each individual, carbon footprint of commuting = distance travelled one way x 2 x 225 (working days per year) x FTE for bathing study period x emission factor for vehicle used. The FTE is described in Table S6.

Total carbon footprint of staff commuting = **10340.23 kg CO_2_e**

Module 3

3.1 Travel to meetings

The majority of meetings were held online via Microsoft Teams. One meeting was held in London as a hybrid meeting, lasting 2.5 hours, with 3 people attending online and 7 people travelling to the venue. Table S8 shows the meeting details and carbon footprint calculations.

Carbon footprint of online meetings:

Number of people x hours x EF for videoconferencing with cameras on (0.1573 kg CO_2_e per person per hour) (10). This is calculated for each meeting individually and then summed.

**Carbon footprint of summed online meetings = 57.23 kg CO_2_e**

Carbon footprint of travel to the meeting held in person:

Number of passengers x distance travelled (km) x EF provided below (p.km measures emissions that are attributed on a single-person basis) (2):

- National rail: 0.044433 kg CO_2_e per p.km.
- London Underground: 0.035082 kg CO_2_e per p.km
- Local bus (not London): 0.147233 kg CO_2_e per p.km
- Average petrol car: 0.209419 kg CO_2_e per km

Carbon footprint of travel to the in-person meeting for in-person attendees:

- Statistician
  - Return journey was same as outbound journey.
  - Home > meeting venue (walked) **0 kg CO_2_e**
- Chair, Clinical epidemiologist
  - Return journey was same as outbound journey.
  - Walked from home to local train station. **0 kg CO_2_e**
  - Local train station > London Waterloo (national rail, 104 km). 104 x 2 x 0.044433 = **9.242064 kg CO_2_e**
  - London Waterloo > Warren Street (London Underground, 3.2 km). 3.2 x 2 x 0.035082 = **0.2245248 kg CO_2_e**
  - Warren Street > meeting venue (walked). **0 kg CO_2_e**
- GP
  - Return journey was same as outbound journey.
  - Home > local Railway Station (car, 4.8 km). 4.8 x 2 x 0.209419 = **2.0104224 kg CO_2_e**
  - Local Railway Station > London Paddington Station (national rail, 251 km). 251 x 2 x 0.044433 = **22.305366 kg CO_2_e**
  - London Paddington Station > meeting venue (walked). **0 kg CO_2_e**
- PPI representative
  - Return journey was same as outbound journey.
  - Home > local train Station (walked). **0 kg CO_2_e**
  - Local train Station > London Kings Cross (national rail, 531 km). 531 x 2 x 0.044433 = **47.187846 kg CO_2_e**
  - London Kings Cross > Warren Street (London underground, 1.8 km). 1.8 x 2 x 0.035082 = **0.1262952 kg CO_2_e**
  - Warren Street > meeting venue (walked). **0 kg CO_2_e**
- Programme Manager
  - Return journey was same as outbound journey.
  - Home > local train station (car, 5.6 km). 5.6 x 2 x 0.209419 = **2.3454928 kg CO_2_e**
  - Local train station > London St Pancras (national rail, 171 km) = 171 x 2 x 0.044433 = **15.196086 kg CO_2_e**
  - London St Pancras > meeting venue (walked). **0 kg CO_2_e**
- PPI lead
  - Home > Local train station (walked). **0 kg CO_2_e**
  - Local train station > London St Pancras (national rail, 172 km). 172 x 0.044433 = **7.642476 kg CO_2_e**
  - London St Pancras > meeting venue (walked). **0 kg CO_2_e**
  - Meeting venue > London St Pancras (walked). **0 kg CO_2_e**
  - London St Pancras > Local train station (national rail, 172 km). 172 x 0.044433 = **7.642476 kg CO_2_e**
  - Local train station > home (bus, 4.7 km). 4.7 x 0.147233 = **0.6919951 kg CO_2_e**
- Chief Investigator
  - Return journey was same as outbound journey.
  - Home > Local train station (walked). **0 kg CO_2_e**
  - Local train station > London St Pancras (national rail, 172 km). 172 x 2 x 0.044433 = **15.284952 kg CO_2_e**
  - London St Pancras > meeting venue (walked). **0 kg CO_2_e**

Total for in person – 129.9 kg CO_2_e

Total kg CO_2_e attributed to both online and in-person meetings = **187.13 kg CO_2_e**

3.2. Sustenance

During the meeting in London, lunch was provided.

Carbon footprint:

Quantity x EF for meeting lunches or hotel dinners (vegetarian) (2.6 kg CO_2_e per meal per person) (11).

Assumptions:

- All members of staff that attended ate one meal.
- All food was vegetarian.

Number of staff that attended = 7

Number of meals per staff = 1

7 x 1 x 2.6 = **18.20 kg CO_2_e**

Module 5

5.1. Data collection and query exchange between CTU and sites

Email traffic:

Emails were sent between participants and staff members via the Rapid Eczema Trials mailbox.

Assumptions:

- Completely accurate data were unattainable because emails relevant to the study would have been sent and received via multiple mailboxes, all of which also have emails unrelated to this study. The most accurate work around we could find was to footprint all of the emails (both relevant and irrelevant to the bathing study) to and from one mailbox - the Rapid Eczema Trials mailbox).

Carbon footprint of email traffic:

Number of emails between 01/01/2023 and 07/11/2024 x EF for emails without (10g CO_2_e per email) and with (20g CO_2_e per email) attachments, respectively (7).

Number of emails sent/received without attachments = 12,047

Number of emails sent/received with attachments = 2771

12,047 x 10 = 120,470 g CO_2_e = 120.47 kg CO_2_e

2771 x 20 = 55,420 g CO_2_e = 55.42 kg CO_2_e

Total carbon footprint of emails = **175.89 kg CO_2_e**

Data collection via electronic trial databases/systems:

In the bathing study, the Redcap database sent out automated emails and captured data from filled in consent forms and questionnaires. Each patient who took part in the bathing study had to complete a baseline questionnaire, which included an e-consent form. Furthermore, patients were asked to complete a questionnaire after each week of the 4 weeks of the study (4 follow-up questionnaires in total per person).

Assumptions:

- Questionnaires were completed on smartphones.

Carbon footprint of data collection via electronic trial databases/systems:

Storage size x number of years x EF for data storage and transmission (0.01724 kg CO_2_e per GB per year) (6)

File size = 1 GB

Number of years stored = 5

1 x 5 x 0.01724 = **0.09 kg CO_2_e**

Carbon footprint of the questionnaires:

(Data storage and transmission EF associated with web surfing [0.009441 kg CO_2_e per hour] (6)+ smartphone EF [0.015068 kg CO_2_e per hour]) (8) x total time taken to complete all questionnaires (hours) by all patients.

Carbon footprint of questionnaires

2 emission factors added together = 0.024509 kg CO_2_e per hour

Time taken to complete baseline questionnaire = 20 mins

Total number of participants = 438

Total time taken to complete baseline questionnaires = 146 hours

Time taken to complete one follow-up questionnaire = 10 mins

Total number of follow up questionnaires completed = 367 (week 1) + 327 (week 2) + 300 (week 3) + 340 (week 4) = 1334

Total time taken to complete all follow up questionnaires = (10 x 1334)/60 = 222.33 hours

Total number of hours taken to complete all questionnaires (baseline and follow up) = 368.33 hours

368.33 x 0.024509 = **9.03 kg CO_2_e**

Total carbon footprint = **185.01 kg CO_2_e**

Module 10

10.1. Storage and archiving of essential trial documentation and data

Electronic documents that were produced across the trial were stored in one of two Microsoft Teams channels: ‘Rapid Eczema Trials’ or ‘Co-Production Groups’.

Carbon footprint of electronic documents produced:

File size (GB) of both channels x duration of data storage x electronic data storage EF (0.01724 kg CO_2_e per GB per year) (6).

Assumptions:

- Both channels contain documents for the whole Rapid Eczema Trials programme, not just the bathing study. It is assumed that 1/3 of the contents are relevant to the bathing study.

Rapid Eczema Trials folder size = 83.1 GB

Co-production groups folder size = 5.1 MB = 0.0051 GB

Total combined folder size = 83.1051 GB

83.1051/3 = 27.7017 GB

Storage time = 5 years

27.7017 x 5 x 0.01724 = **2.39 kg CO_2_e**

Table S1: The number of A4 posters and A5 leaflets sent to each GP surgery, and the distances between NCTU and each GP surgery.

| Practice name | No. A4 posters sent | No. A5 leaflets sent | Distance (km) between NCTU and GP |
| --- | --- | --- | --- |
| Leen View Surgery | 4 | 10 | 8.20765 |
| Village Health Group | 4 | 0 | 13.3576 |
| Saxon Cross Surgery | 2 | 2 | 6.92018 |
| Churchfields Medical Practice | 2 | 10 | 6.11551 |
| Pinfold Medical Practice | 4 | 10 | 24.1402 |
| Chilwell Valley and Meadows Practice | 2 | 0 | 7.88579 |
| Lindum Medical Practice | 2 | 10 | 70.8111 |
| King's Medical Centre | 6 | 10 | 28.9682 |
| Nettleham Medical Practice | 2 | 0 | 77.2485 |
| Forest Medical | 2 | 2 | 28.9682 |
| Billesdon Surgery | 2 | 10 | 54.7177 |
| South Leics Med Group | 6 | 10 | 64.3738 |
| St Georges Medical Practice | 2 | 0 | 6.92018 |
| Plains View Surgery | 5 | 5 | 8.36859 |
| Langham Place Surgery | 4 | 20 | 101.389 |
| Moulton Surgery | 2 | 0 | 106.217 |
| Rivergreen Medical Centre | 2 | 10 | 7.40298 |
| Albany House Medical Centre | 2 | 10 | 114.263 |
| Vine House Surgery | 2 | 10 | 41.8429 |
| St Johns Surgery | 2 | 2 | 46.671 |
| Colsterworth Medical Centre | 2 | 4 | 56.327 |
| Billinghay Medical Practice | 2 | 4 | 85.2952 |
| Sleaford Medical Group | 2 | 4 | 72.4205 |
| Glenside Country Practice | 2 | 4 | 64.3738 |
| Millview Medical Centre | 2 | 10 | 82.0765 |
| Danes Camp Medical Centre | 2 | 0 | 106.217 |
| Caythorpe and Ancaster Surgery | 2 | 4 | 61.1151 |
| The Family Medical Centre | 3 | 10 | 6.75924 |
| Hill View Surgery | 2 | 10 | 27.3588 |
| Parkside Medical Practice | 4 | 40 | 7.56392 |
| Long Bennington Surgery | 2 | 4 | 41.8429 |
| The Manor Surgery | 2 | 10 | 5.31084 |
| St Peter Hill Surgery | 2 | 10 | 45.0616 |
| Harrowby Lane Surgery | 2 | 4 | 46.671 |
| Market Cross Surgery | 2 | 4 | 64.3738 |
| New Springwells Surgery | 2 | 10 | 72.4205 |
| Ruskington Surgery | 2 | 2 | 72.4205 |
| Derby Road Health Centre | 2 | 2 | 2.09215 |
| James Alexander Family Practice | 6 | 50 | 146.45 |
| Conisbrough Group Practice | 2 | 10 | 70.8111 |
| Ampleforth & Hovingham Surgeries | 2 | 30 | 180.247 |
| Ayton & Snainton Medical Practice | 3 | 30 | 197.949 |
| Springbank Surgery | 4 | 20 | 141.622 |
| Gibson Lane Practice | 3 | 150 | 114.263 |
| The Park Surgery | 5 | 50 | 185.075 |
| Woodstock Bower Group Practice | 3 | 10 | 67.5924 |
| Ancora medical practice* | 2 | 2 | 106.217 |
| Ashby Turn Primary Care Centre* | 2 | 2 | 106.217 |
| Cambridge Avenue Medical Centre* | 2 | 2 | 109.435 |
| Cedar Medical Practice* | 2 | 2 | 109.435 |
| Kirton Lindsay & Scotter Surgery* | 2 | 2 | 96.5606 |
| West Common Lane Teaching practice* | 2 | 2 | 114.263 |
| Posterngate Surgery | 2 | 20 | 119.091 |
| Kirkburton Health Centre | 2 | 20 | 102.998 |
| Allerton Medical Centre | 20 | 200 | 120.701 |
| Stockwell Road Surgery | 5 | 20 | 143.232 |
| The White House Surgery | 2 | 12 | 62.7644 |
| Bartholomew Medical Group | 2 | 5 | 107.826 |
| Littlefield Surgery | 6 | 6 | 122.31 |
| MyHealth Group | 4 | 0 | 154.497 |
| Market Weighton | 1 | 5 | 140.013 |
| Mosborough Health Centre | 5 | 30 | 51.499 |
| Pickering Medical Practice | 6 | 50 | 183.465 |
| Clover Group Practice** | 2 | 10 | 65.9831 |
| Buchanan Road Surgery** | 2 | 10 | 70.8111 |
| Clover City Practice** | 2 | 10 | 70.8111 |
| Heeley Green Surgery** | 2 | 10 | 59.5457 |
| Sothall & Beighton Health Centres** | 2 | 10 | 53.1084 |
| Harold Street Medical Centre** | 2 | 10 | 72.4205 |
| North Darnall Health Centre** | 2 | 10 | 67.5924 |
| Crookes Practice** | 2 | 10 | 62.7644 |
| Ecclesfield Group Practice** | 2 | 10 | 74.0298 |
| Greystones Medical Centre** | 2 | 10 | 61.1551 |
| Nethergreen Surgery** | 2 | 10 | 62.7644 |
| Wincobank Medical Centre** | 2 | 10 | 67.5924 |
| Oaklands Health Centre | 2 | 100 | 102.998 |
| **TOTAL** | **219** | **1217** | **5690.59933** |

*Surgeries part of the North Lincolnshire South PCN Group.

**Surgeries part of the Primary Care Sheffield Cluster.

Table S2 The number of GP letter invites sent to patients

| Practice name | Adult invitations | Child invitations | Total letter invites sent |
| --- | --- | --- | --- |
| Billesdon Surgery | 303 | 90 | 393 |
| South Leics Med Group | 721 | 297 | 1018 |
| **Total** |  |  | **1411** |

Table S3 – total number of text messages sent to participants, as part of recruitment process.

| Practice name | Adult invitations | Child invitations | Follow up sent? | Total adult invitations (initial plus follow-up) | Total child invitations (initial plus follow-up) | Total text messages sent |
| --- | --- | --- | --- | --- | --- | --- |
| Leen View Surgery | 536 | 280 | No | 536 | 280 | 816 |
| Village Health Group | 188 | 58 | No | 188 | 58 | 246 |
| Saxon Cross Surgery | 416 | 72 | No | 416 | 72 | 488 |
| Churchfields Medical Practice | 346 | 155 | No | 346 | 155 | 501 |
| Pinfold Medical Practice | 144 | 123 | No | 144 | 123 | 267 |
| Chilwell Valley and Meadows Practice | 572 | 172 | No | 572 | 172 | 744 |
| Lindum Medical Practice | 103 | 247 | No | 103 | 247 | 350 |
| King's Medical Centre | 240 | 137 | No | 240 | 137 | 377 |
| Nettleham Medical Practice | 595 | 51 | No | 595 | 51 | 646 |
| Forest Medical | 462 | 202 | No | 462 | 202 | 664 |
| St Georges Medical Practice | 641 | 62 | No | 641 | 62 | 703 |
| Plains View Surgery | 375 | 78 | No | 375 | 78 | 453 |
| Langham Place Surgery | 173 | 93 | No | 173 | 93 | 266 |
| Moulton Surgery | 391 | 111 | No | 391 | 111 | 502 |
| Rivergreen Medical centre | 265 | 111 | No | 265 | 111 | 376 |
| Albany House Medical Centre | 674 | 86 | No | 674 | 86 | 760 |
| Vine House Surgery | 404 | 71 | No | 404 | 71 | 475 |
| St Johns Surgery | 345 | 2 | No | 345 | 2 | 347 |
| Colsterworth Medical Centre | 61 | 23 | No | 61 | 23 | 84 |
| Billinghay Medical Practice | 190 | 34 | No | 190 | 34 | 224 |
| Sleaford Medical Group | 586 | 190 | No | 586 | 190 | 776 |
| Glenside Country Practice | 146 | 40 | No | 146 | 40 | 186 |
| Millview Medical Centre | 363 | 126 | No | 363 | 126 | 489 |
| Caythorpe and Ancaster Surgery | 466 | 107 | No | 466 | 107 | 573 |
| The Family Medical Centre | 210 | 72 | No | 210 | 72 | 282 |
| Parkside Medical Practice | 189 | 281 | No | 189 | 281 | 470 |
| Long Bennington Surgery | 248 | 40 | No | 248 | 40 | 288 |
| The Manor Surgery | 506 | 167 | No | 506 | 167 | 673 |
| St Peter Hill Surgery | 681 | 162 | No | 681 | 162 | 843 |
| Harrowby Lane Surgery | 157 | 69 | No | 157 | 69 | 226 |
| Market Cross Surgery | 211 | 39 | No | 211 | 39 | 250 |
| New Springwells Surgery | 203 | 46 | No | 203 | 46 | 249 |
| Ruskington Surgery | 208 | 64 | No | 208 | 64 | 272 |
| Derby Road Health Centre | 373 | 135 | No | 373 | 135 | 508 |
| James Alexander Family Practice | 52 | 36 | yes | 104 | 72 | 176 |
| Conisbrough Group Practice | 389 | 167 | yes | 778 | 334 | 1112 |
| Ampleforth & Hovingham Surgeries | 185 | 37 | yes | 370 | 74 | 444 |
| Ayton & Snainton Medical Practice | 454 | 41 | yes | 908 | 82 | 990 |
| Springbank Surgery | 347 | 118 | yes | 694 | 236 | 930 |
| Gibson Lane Practice | 162 | 578 | yes | 324 | 1156 | 1480 |
| The Park Surgery | 366 | 100 | no | 366 | 100 | 466 |
| Woodstock Bower Group Practice | 375 | 144 | yes | 750 | 288 | 1038 |
| North Lincolnshire South PCN (6 practices) | 1849 | 791 | no | 1849 | 791 | 2640 |
| Posterngate Surgery | 465 | 165 | yes | 930 | 330 | 1260 |
| Allerton Medical Centre | 421 | 163 | yes | 842 | 326 | 1168 |
| Bartholomew Medical Group | 463 | 241 | no | 463 | 241 | 704 |
| Market Weighton | 337 | 113 | yes | 674 | 226 | 900 |
| Mosborough Health Centre | 388 | 24 | yes | 776 | 48 | 824 |
| Pickering Medical Practice | 160 | 0 | no | 160 | 0 | 160 |
| PCS Cluster (12 practices) | 2568 | 1177 | no | 2568 | 1177 | 3745 |
| **TOTAL** |  |  |  |  |  | **33411** |

Table S4 Facebook ad information

| Date ad completed | No. of views | File size (mb) | No. of views x file size (mb) | No. of views x file size (gb) |
| --- | --- | --- | --- | --- |
| 05/02/2024 | 68635 | 0.904 | 62,046.04mb | 62.04604 |
| 21/02/2024 | 32092 | 2.27 | 72,848.84 | 72.84884 |
| 26/02/2024 | 330500 | 2.6 | 859,300 | 859.3 |
| 23/04/2024 | 51023 | 0.904 | 46,124.792 | 46.124792 |
| 03/05/2024 | 45063 | 2.27 | 102,293.01 | 102.29301 |
| 04/06/2024 | 28053 | 0.314 | 8,808.642 | 8.808642 |
| 25/06/2024 | 19596 | 74.7 | 1,463,821.2 | 1463.821 |

Table S5: Webpage information and calculations. Webpages without videos are highlighted in orange.

| Webpage | g CO2e per view | No. of views | g CO2e per page  (g CO2e per view x number of views) | Video length (sec) | No. of times video watched | Video watch time (h) = video length (sec) x no. of times watched / 3600 | Websurfing footprint |
| --- | --- | --- | --- | --- | --- | --- | --- |
| Daily bathing child | 0.44 | 64 | 28.16 | 166 | 7 | 0.32277778 |  |
| Daily bathing adult | 0.43 | 207 | 89.01 | 145 | 24 | 0.96666667 |  |
| Weekly bathing child | 0.44 | 122 | 53.68 | 233 | 9 | 0.5825 |  |
| Weekly bathing adult | 0.45 | 204 | 91.8 | 220 | 19 | 1.16111111 |  |
| More info (adult) | 0.44 | 1,142 | 502.48 | 205 | 666 | 37.925 |  |
| More info (child) | 0.43 | 547 | 235.21 | 104 | 322 | 9.30222222 |  |
| Homepage for bathing study | 0.43 | 22,470 | 9662.1 | 269 | 32 | 2.39111111 |  |
|  |  |  |  |  |  |  | 1.29 kg CO_2_e (for all webpages with videos) |
| GDPR page | 0.12 | 29 | 3.48 | N/A | N/A | N/A |  |
| Blog about bathing habits | 0.13 | 74 | 9.62 | N/A | N/A | N/A |  |
| Blog about bathing and eczema | 0.31 | 117 | 36.27 | N/A | N/A | N/A |  |
| Research summary about bathing and eczema | 0.12 | 44 | 5.28 | N/A | N/A | N/A |  |
| Bathing results webinar page | 0.12 | 19 | 2.28 | N/A | N/A | N/A |  |
|  |  |  |  |  |  |  | 0.23 kg CO_2_e (for all webpages without videos) |
|  |  |  | 10,717.09 total |  |  | 52.6513889 hours watch time - videos |  |

Table S6: FTE information and calculations. All staff up to and including Staff 11 worked on trial delivery. All staff from Staff 12 onwards worked on trial design.

| Name | Start date | End date | FTE | %FTE on bathing trial | FTE on bathing trial per year = FTE x % FTE on bathing trial | Number of days in period being footprinted | FTE on bathing trial for period being footprinted |
| --- | --- | --- | --- | --- | --- | --- | --- |
| Staff 1 | 01/05/23 | 07/11/24 | 0.6 | 100 | 0.6 | 557 | 0.92 |
| Staff 2 | 01/05/23 | 07/11/24 | 0.6 | 100 | 0.6 | 557 | 0.92 |
| Staff 3 | 01/05/23 | 07/11/24 | 0.125 | 100 | 0.125 | 557 | 0.19 |
| Staff 4 | 01/05/23 | 07/11/24 | 0.1 | 100 | 0.1 | 557 | 0.15 |
| Staff 5 | 01/09/23 | 07/11/24 | 0.4 | 100 | 0.4 | 434 | 0.48 |
| Staff 6 | 01/05/23 | 07/11/24 | 0.05 | 100 | 0.05 | 557 | 0.08 |
| Staff 7 | 01/05/23 | 07/11/24 | 0.2 | 100 | 0.2 | 557 | 0.31 |
| Staff 8 | 13/10/23 | 07/11/24 | 0.4 | 100 | 0.4 | 392 | 0.43 |
| Staff 9 | 01/05/23 | 07/11/24 | 0.05 | 100 | 0.05 | 557 | 0.08 |
| Staff 10 | 01/05/23 | 07/11/24 | 0.05 | 100 | 0.05 | 557 | 0.08 |
| Staff 11 | 01/06/24 | 07/11/24 | 0.4 | 100 | 0.4 | 160 | 0.18 |
| Staff 12 | 01/01/23 | 29/01/24 | 0.2 | 40 | 0.08 | 394 | 0.09 |
| Staff 13 | 01/03/23 | 29/01/24 | 1 | 40 | 0.4 | 335 | 0.37 |
| Staff 14 | 01/01/23 | 29/01/24 | 0.4 | 5 | 0.02 | 394 | 0.02 |
| Staff 15 | 01/04/23 | 29/01/24 | 0.6 | 50 | 0.3 | 304 | 0.36 |
| Staff 16 | 01/01/23 | 29/01/24 | 0.5 | 50 | 0.25 | 394 | 0.27 |
| Staff 17 | 01/01/23 | 29/01/24 | 0.5 | 10 | 0.05 | 394 | 0.05 |
| Staff 18 | 01/01/23 | 29/01/24 | 1 | 15 | 0.15 | 394 | 0.16 |
| Staff 19 | 01/09/23 | 29/01/24 | 0.3 | 50 | 0.15 | 151 | 0.12 |
| Staff 20 | 01/01/23 | 29/01/24 | 0.7 | 40 | 0.28 | 394 | 0.30 |
| Staff 21 | 01/01/23 | 29/01/24 | 0.4 | 5 | 0.02 | 335 | 0.02 |
|  |  |  |  |  |  |  | Total = 5.58 |

Table S7 Staff commuting information, including carbon footprint of commuting for study period

| Name | Commuting method | Distance (single) from home to office (km) | Emission factor (2) | Carbon footprint of commute (kg CO_2_e) |
| --- | --- | --- | --- | --- |
| Staff 1 | Petrol car | 10.6 | 0.209419 | 919.01 |
| Staff 2 | Petrol car | 67.6 | 0.209419 | 5869.55 |
| Staff 3 | Petrol car | 77.2 | 0.209419 | 1382.29 |
| Staff 4 | Cycle | 3.21 | 0 | 0 |
| Staff 5 | Tram | 6.76 | 0.03693 | 53.92 |
| Staff 6 | Electric car | 25.7 | 0.05794 | 53.61 |
| Staff 7 | Bus | 8.05 | 0.147233 | 165.34 |
| Staff 8 | Tram | 4.83 | 0.03693 | 34.51 |
| Staff 9 | Petrol car | 7.40 | 0.209419 | 55.79 |
| Staff 10 | Tram | 3.21 | 0.03693 | 4.27 |
| Staff 11 | Petrol car | 7.24 | 0.209419 | 122.81 |
| Staff 12 | Bus | 7.89 | 0.147233 | 47.05 |
| Staff 13 | Tram | 5.79 | 0.03693 | 35.60 |
| Staff 14 | Petrol car | 9.01 | 0.209419 | 16.98 |
| Staff 15 | Bus | 9.17 | 0.147233 | 218.72 |
| Staff 16 | Cycle | 1.61 | 0 | 0 |
| Staff 17 | Cycle | 4.02 | 0 | 0 |
| Staff 18 | Cycle | 4.02 | 0 | 0 |
| Staff 19 | Bus | 13.7 | 0.147233 | 108.92 |
| Staff 20 | Petrol car | 43.5 | 0.209419 | 1229.81 |
| Staff 21 | Petrol car | 11.7 | 0.209419 | 22.05 |

Table S8 - Meetings that took place during the footprinting period, and carbon footprint of online meetings

| Meetings | Number of attendees | Meeting length | % estimated time spent on the bathing study | Online or in person? | Carbon footprint of online meetings = number of attendees x hours spent on bathing study x EF (0.1573) (kgCO_2_e) (10) |
| --- | --- | --- | --- | --- | --- |
| Co-production group prioritisation | 18 | 2 hours | 80% | Online | 4.53 |
| Co-production group prioritisation | 16 | 2 hours | 80% | Online | 4.03 |
| Co-production group trial development | 16 | 2 hours | 50% | Online | 2.52 |
| Co-production group trial development | 16 | 2 hours | 70% | Online | 3.52 |
| Co-production group trial development | 14 | 2 hours | 70% | Online | 3.08 |
| Co-production group trial development | 19 | 2 hours | 90% | Online | 5.38 |
| Co-production group trial development | 9 | 2 hours | 90% | Online | 2.55 |
| Co-production group intervention | 14 | 2 hours | 40% | Online | 1.76 |
| Co-production group intervention | 15 | 2 hours | 80% | Online | 3.78 |
| Co-production group intervention | 19 | 2 hours | 90% | Online | 5.38 |
| Co-production group intervention | 14 | 2 hours | 60% | Online | 2.64 |
| Programme Steering Committee | 8 | 1.5 hours | 40% | Online | 0.76 |
| Programme Steering Committee | 10 | 2.5 hours | 30% | Hybrid (3 online, 7 in-person) | 0.35 |
| Programme Steering Committee | 10 | 1.5 hours | 25% | Online | 0.59 |
| Blind data review meeting | 17 | 2 hours | 100% | Online | 5.35 |
| Trial results reveal meeting | 35 | 2 hours | 100% | Online | 11.01 |

Table S9 - Speed of recruitment calculations for the bathing study and the other NIHR-funded eczema treatment trials

| Trial | Number of participants recruited | Recruitment period | Number of months in recruitment period | Calculation | Speed of recruitment (participants per month) |
| --- | --- | --- | --- | --- | --- |
| Eczema Bathing Study | 438 | 29/1/2024 – 8/7/2024 | 5 | 438/5 | 87.60 |
| BEEP | 1394 | 19/11/2014 – 14/7/2016 | 20 | 1394/20 | 69.70 |
| BEE | 550 | 19/1/2018 – 31/10/2019 | 21 | 550/21 | 26.19 |
| BATHE | 483 | X/11/2014 – X/5/2016 | 18 | 483/18 | 26.83 |
| CLOTHES | 300 | 26/11/2013 – 5/5/2015 | 17 | 300/17 | 17.65 |
| SWET | 336 | X/5/2007 – X/6/2009 | 25 | 336/25 | 13.44 |
| TREAT | 103 | 26/5/2016 – 5/2/2019 | 32 | 103/32 | 3.22 |
| CREAM | 113 | 16/7/2013 – 28/11/2014 | 16 | 113/16 | 7.06 |
| ECO 1 | 340 | 2/12/2019 – 8/12/2020 (2 months paused due to COVID) | 12 – 2 = 10 | 340/10 | 34.00 |
| ECO 2 | 337 | 2/12/2019 – 8/12/2020 (2 months paused due to COVID) | 12 – 2 = 10 | 337/10 | 33.70 |

# **References**

1. TMRP. Enabling lower carbon clinical trials (CiCT) project [Available from: <https://www.methodologyhubs.mrc.ac.uk/about/working-groups/trial-conductwg/tcwg-subgroup-greener-trials/enabling-lower-carbon-clinical-trials-cict-project>.

2. gov.uk. Greenhouse gas reporting: conversion factors 2023 2023 [Available from: <https://www.gov.uk/government/publications/greenhouse-gas-reporting-conversion-factors-2023>.

3. Ecoinventsupport. Ecoinvent version 2 2024 [Available from: <https://support.ecoinvent.org/ecoinvent-version-2>.

4. Willems S, Peersman W, De Maeyer P, Buylaert W, De Maeseneer J, De Paepe P. The impact of neighborhood deprivation on patients’ unscheduled out-of-hours healthcare seeking behavior: a cross-sectional study. BMC family practice. 2013;14(1):136-.

5. Berners-Lee M. How bad are bananas? : the carbon footprint of everything. London: Profile Books; 2020.

6. Obringer R, Rachunok B, Maia-Silva D, Arbabzadeh M, Nateghi R, Madani K. The overlooked environmental footprint of increasing Internet use. Resources, conservation and recycling. 2021;167:105389.

7. mail.com. What's the carbon footprint of an email? 2023 [Available from: <https://www.mail.com/blog/posts/email-carbon-footprint/9/>.

8. Manne S. Examining the carbon footprint of devices 2020 [Available from: <https://devblogs.microsoft.com/sustainable-software/examining-the-carbon-footprint-of-devices/>.

9. HomesAndCommunitiesAgency. Employment density guide 3rd edition 2015 [Available from: <https://www.kirklees.gov.uk/beta/planning-policy/pdf/examination/national-evidence/NE48_employment_density_guide_3rd_edition.pdf>.

10. Wiles K. Turn off that camera during virtual meetings, environmental study says 2021 [Available from: <https://www.purdue.edu/newsroom/archive/releases/2021/Q1/turn-off-that-camera-during-virtual-meetings,-environmental-study-says.html>.

11. WWF. Food in a warming world 2018 [Available from: <https://www.wwf.org.uk/sites/default/files/2018-03/Food_in_a_warming_world_report.PDF>.
